# A Review of Mixed Strain *Clostridium difficile* Colonization and Infection

**DOI:** 10.3389/fmicb.2019.00692

**Published:** 2019-04-10

**Authors:** Pete Dayananda, Mark H. Wilcox

**Affiliations:** ^1^Department of Microbiology, Leeds Teaching Hospitals NHS Trust, Leeds, United Kingdom; ^2^Leeds Institute of Biomedical and Clinical Sciences, University of Leeds, Leeds, United Kingdom

**Keywords:** carriage, competition, microbiome, antibiotic, colon

## Abstract

Given that *Clostridium difficile* is not part of the normal human microbiota, if multiple strains are to accumulate in the colon implies successive exposure events and/or persistent colonization must occur. Evidence of *C. difficile* infection (CDI) with more than one strain was first described in 1983. Despite the availability of increasingly discriminatory bacterial fingerprinting methods, the described rate of dual strain recovery in patients with CDI has remained stable at ∼5–10%. More data are needed to determine when dual strain infection may be harmful. Notably, one strain may block the establishment of and infection by another. In humans, patients colonized by non-toxigenic *C. difficile* strain are at a lower risk of developing CDI. Further studies to elucidate the interaction between co-infecting or colonizing and infecting *C. difficile* strains may help identify potential exploitable mechanisms to prevent CDI.

## Introduction

*Clostridium (Clostridioides) difficile* is an anaerobic, spore forming, Gram-positive bacillus (Martin et al., 2016). Historically, *C. difficile* infection (CDI) primarily occurs in hospitalized patients secondary to antibiotic use (Kelly and LaMont, 1998). However, in the past decade, the proportion of *C. difficile* infection occurring in the community (previously thought to be low risk) is increasing (DePestel and Aronoff, 2013). This highlights the endemicity of *C. difficile* outside healthcare institutions. In addition, the emergence of a more virulent *C. difficile* strain BI/NAP1/027 changed perceptions of *C. difficile* from an easily treated side-effect of antibiotic use to a leading cause of infectious diarrhea with increased morbidity and mortality worldwide (Baines et al., 2013; Ghose, 2013; Sun et al., 2016).

There are over 800 recognized strain types (ribotypes) of *C. difficile* and only toxin-producing strains are associated with disease (Tonna and Welsby, 2005; Vedantam et al., 2012). *C. difficile* associated diarrhea is mediated by the production of toxin A (TcdA) and toxin B (TcdB) released into the gut as a result of colonization by toxigenic strains (van den Berg et al., 2005). Early hamster models suggested that TcdA is a key determinant of intestinal inflammation (Lyerly et al., 1985), but subsequent studies suggested that TcdB is more potent (Savidge et al., 2003; Lyras et al., 2009). Furthermore, a monoclonal anti-toxin B antibody, but not an anti-toxin A antibody, was effective at reducing recurrence in patients treated for CDI (Wilcox et al., 2017). Also, there is a growing body of evidence for TcdA negative/TcdB positive CDI cases (Samra et al., 2002; Carter et al., 2015; Di Bella et al., 2016). Therefore, the importance of TcdA in human beings remains uncertain and needs further clarification. Since the emergence of the more virulent BI/NAP1/027 strain of *C. difficile*, the role of a third binary toxin, *C. difficile* transferase (CDT) is increasingly recognized for its association with enhanced virulence and higher patient mortality (Gerding et al., 2014; Berry et al., 2017).

The method used for diagnosing CDI is one of the factors limiting detection of multiple *C. difficile* strains in health or disease. CDI diagnosis is based ideally on detection of free fecal toxin, or, with less specificity, by the presence of toxin genes (Surawicz et al., 2013; Martin et al., 2016). Therefore, in the majority of settings, *C. difficile* culture is not performed and so the number of *C. difficile* strains present is not determined.

The likelihood of detecting multi-strain infection or colonization varies with the methods used. Multiple strains can be detected by methods that can distinguish individual strains. In the context of *C. difficile*, these methods include restriction enzyme analysis (REA), pulsed field gel electrophoresis (PGFE), PCR ribotyping, multilocus variable number tandem repeat analysis (MLVA), multilocus sequence typing (MLST), and whole genome sequencing (WGS) (Tenover et al., 2011; Knetsch et al., 2013; Sim et al., 2017). Multiple strains may be detected by the presence of more alleles present at a particular locus than is possible if just one strain is present, dissimilar genotypes from different colonies grown from the same isolate or difference in ability to produce cytotoxin (Borriello and Honour, 1983; Balmer and Tanner, 2011). Notably, the limited studies that have investigated the presence of multiple strains of *C. difficile* in patients with CDI, differ in their case selection criteria and in the methods used for *C. difficile* culture, the number of colonies tested and differentiation of strains (often reflecting the available diagnostic technology) (Table 1).

**Table 1:** Summary of studies exploring the presence of dual/mixed strain *Clostridium difficile* infection.

Author + year	Isolate selection	*C. difficile* identification	Number of cases	Culture method	No. of colonies tested per case	*C. difficile* strain differentiation	Cases with more than 1 *C. difficile* strain
Borriello and Honour, 1983	6 patients with antibiotic associated diarrhea and 1 with diarrhea unrelated to antibiotic use	*C. difficile* analytical profile index (API). Growth of isolates inhibited by *Clostridium beijerinckii*	7	Cycloserine cefoxitin agar.	At least 10	Examined for production of cytotoxin using MRC-5 and VERO cells. Identified toxin producing and non-producing strains	7
Sharp and Poxton, 1985	4 strains of stored *C. difficile* isolates	Unspecified	3	Cycloserine cefoxitin fructose agar (CCFA)	8	Ethylenediaminetetraacetic acid (EDTA) antigen extraction and immunoblotting	2
Devlin et al., 1987	110 stored clinical isolates	Unspecified	110	Cycloserine-cefoxitin-fructose agar with sodium taurocholate (TCCFA).	Approximately 10	Plasmid profile and restriction enzyme analysis (REA)	0
O’Neill et al., 1991	10 patients with >1 isolation of *C. difficile*	Latex particle agglutination test	10	CCFA	10	REA Examined for production of cytotoxin using VERO cells	0
Wilcox et al., 1998	Frozen isolates from patients with >2 *C. difficile* cytotoxin positive fecal samples	Colonial morphology, odor and RapID ANA II	27	Cycloserine, cefoxitin, egg yolk agar	1–2	Random amplified polymorphic DNA (RAPD)	0
van den Berg et al., 2005	Frozen isolates from patients with first episodes of *C. difficile* associated diarrhea	Enzyme linked fluorescence assay and culture	23	Columbia agar with colistin + nalidixic acid and/or *C. difficile* agar with moxalactam and cysteine.	Approximately 5	PCR ribotyping, toxinogenicity and clindamycin resistance	2
Tanner et al., 2010	Pre-selected from a pool of cases typed as 027 strain	PCR ribotyping	39	CCFA	5	Multilocus variable number tandem repeat analysis (MLVA)	5
Kamboj et al., 2011	Patients with 2 episodes of *C. difficile* infection occurring >2 weeks apart identified using microbiology records	*C. difficile* neutralization assay and enzyme immunoassay (EIA) for glutamate dehydrogenase (GDH)	102	Unspecified	Unspecified	PCR ribotyping	0
Hell et al., 2011	Patients with nosocomial *C. difficile* chosen arbitrarily	Enzyme linked immunosorbent assay (ELISA) for *C. difficile* TcdA and TcdB	11	Cycloserine/cefoxitin agar	5	PCR ribotyping	1
Eyre et al., 2012	Paired stool samples from patients collected on the same day	EIA for GDH and culture	305	Unspecified	Unspecified	Multilocus sequence typing (MLST)	21
Behroozian et al., 2013	Stored isolates from *C. difficile* infected patients	3 step diagnostic algorithm: (1) EIA for GDH; (2) EIA for *C. difficile* toxin A and/or B; (3) PCR for presence of tcdB gene	102	Pre-reduced, TCCFA	95	PCR ribotyping	16
Sun et al., 2016	Patients with >1 episode of CDI occuring at least 8 weeks apart	Stool sample positive by PCR for *C. difficile*.	52	Not specified	5	MLST	4


## Significance

A key issue regarding colonization or infection by more than one strain of *C. difficile* is the determination of whether recurrence is due to the same (relapse) or different (reinfection) strain. Most such CDI recurrence studies have examined single or few colonies, or have not used a highly discriminative method, both of which reduce the chance of isolating multiple strains of *C. difficile*. For example, Figueroa et al. (2012) examined 90 patients who had recurrent *C. difficile* infection using REA on single *C. difficile* colonies (Figueroa et al., 2012). They showed that 75 participants (83.3%) had a relapse. The remaining 15 (16.7%) participants were found to have a reinfection. Based on the results obtained, there was no evidence to suggest the presence of concomitant carriage of more than 1 strain of *C. difficile* (Figueroa et al., 2012) Although REA has respectable discriminatory power, newer methods such as capillary PCR ribotyping and MLVA are more discriminatory and therefore have a higher chance of identifying mixed *C. difficile* strain infections (Kuijper et al., 2009). Kamboj et al. (2011) used PCR ribotyping to explore patients with recurrent *C. difficile* infection. The results suggested that the majority of patients with recurrent *C. difficile* infection within 8 weeks (85/102 patients) had a relapse and not a reinfection. This method has similar discriminative power to REA (Kuijper et al., 2009). Based on the results, there was no evidence to suggest the presence of more than 1 strain of *C. difficile* at any one time (Kamboj et al., 2011). This may be due to the small number of colonies studied as per the published protocol (Bidet et al., 1999). A recent study by Behroozian et al. (2013) recognized this limitation and analyzed approximately 95 colonies per sample and found evidence of more than 1 *C. difficile* ribotype in 16/102 (16%) cases. Even with the large number of colonies studied, there is a chance that less abundant ribotypes will be overlooked (Behroozian et al., 2013).

As *C. difficile* is ubiquitous in nature, the presence of multiple strains could simply reflect a recently ingested strain when another has already colonized or been newly ingested. However, colonization by multiple strains could affect the level of host protection against *C. difficile* infection. At present, there is insufficient evidence to determine the full implications of colonization/infection with more than 1 strain of *C. difficile*. Baines et al. (2013) demonstrated using an *in vitro* gut model that 2 different population of *C. difficile* (differentiated by antimicrobial susceptibility) were able to concurrently colonize, populate and produce toxin. It is however, not possible to determine the degree of contribution toward toxin production (Baines et al., 2013). Longitudinal studies are needed to determine the significance of this phenomenon in human beings.

Data from murine models suggest that colonization with a non-toxigenic strain of *C. difficile* protects against disease in hamster following a challenge with a toxigenic strain (Merrigan et al., 2009). Balmer et al. suggests that in human beings, infection with more than 1 strain of *C. difficile* is likely to be a significant clinical and immunological phenomenon as it may overwhelm the immune system by influencing the host immune response in different ways. This may also affect pathogen evolution, potentiate competitive, or mutualistic pathogen-pathogen interaction, horizontal gene flow and treatment options compared with single strain infection (Balmer and Tanner, 2011). On the other hand, the presence of two competing strains can be beneficial to the host as they could control each other, similar to how probiotics have been proposed for the prevention of CDI (Goldenberg et al., 2013). Notably, Gerding et al. demonstrated that patients with an episode of primary CDI or first recurrence within 8 weeks of the primary episode benefitted from colonization with non-toxigenic *C. difficile* strain M3 (NTCD-M3). The recurrence rate of CDI in patients colonized with NTCD-M3 was significantly lower compared with patients who were not (11 vs. 30%). This reaffirms the notion that colonization with a non-toxigenic strain is beneficial to the host (Gerding et al., 2015).

## Dual Strain Infection

The observed incidence of mixed *C difficile* toxigenic strain infection has been relatively stable over recent decades at approximately 7–16% of all cases (Eyre et al., 2012, 2013; Behroozian et al., 2013; Sun et al., 2016). Evidence of mixed strain infection was first reported by Borriello and Honour (1983). The authors found that stool samples from all 6 studied cases showed isolates of *C. difficile* that differed in cytotoxin production (Borriello and Honour, 1983). O’Neill et al. (1991) later reported a patient who had suffered both a reinfection and relapse. The isolated strain did not produce cytotoxin *in vitro* but toxin was detected in the stools. They hypothesized that this is due to the presence of concomitant strains of *C. difficile.* However, further investigation on 10 different colonies from each sample using REA and cytotoxin studies did not support this (O’Neill et al., 1991). Similarly, in a larger study, Wilcox et al. (1998) retrospectively analyzed *C. difficile* colonies using random amplification of polymorphic DNA (RAPD) fingerprinting, but did not identify show more than one *C. difficile* strain per sample time point (Wilcox et al., 1998). This may be due to the fact that mixed infection is a rare occurrence or due to the limitations of the detection methods (Barbut et al., 2000; Behroozian et al., 2013). More recently, Hell et al. (2011); van den Berg et al. (2005) investigated 5 or fewer colonies from each sample and found that 1/11 (9.1%) and 2/23 (8.7%) of samples, respectively, had multiple strains of *C. difficile*.

With advancing technology, improved sampling methods and sophisticated genotyping, *C. difficile* transmissions leading to mixed infection and the presence of different toxigenic *C. difficile* strains are being more readily identified (Eyre et al., 2012, 2013). However, the reported rate of mixed infection remains similar.

## Conclusion

Knowledge and understanding of *C. difficile* has grown considerably since George and colleagues made the link between *C. difficile* and human diseases (Heinlen and Ballard, 2010). Evidence of dual strain infection was first identified by Borriello and Honour (1983) after observing differential expression of toxins (Borriello and Honour, 1983). With advancing technology, *C. difficile* typing methods are becoming more discriminative and therefore mixed strain infection can be detected more readily. Interestingly, the incidence of mixed strain *C. difficile* infection has been stable.

Currently, there are no studies examining the role of mixed or dual strain infection with *C. difficile* in human beings. A previous study in hamsters suggests that mixed strain infection has the potential to be both beneficial and harmful depending on the nature of the infecting strains (Merrigan et al., 2009). In human beings, it is recognized that patients who are colonized by non-toxigenic *C. difficile* strain is at lower risk of developing *C. difficile* infection while in hospital. This is supported by results from a Phase 2 randomized controlled trial using non-toxigenic *C. difficile* spores to prevent recurrent *C. difficile* infection (Gerding et al., 2015). Further studies to elucidate the interaction between co-infecting or colonizing and infecting *C. difficile* strains may help identify potential exploitable mechanisms to prevent *C. difficile* infection.

## Author Contributions

MW wrote the manuscript scope and outline, supervised the manuscript writing, and carried out multiple rounds of editing. PD carried out the literature search, wrote the manuscript draft, and carried out multiple rounds of re-writing.

## Conflict of Interest Statement

MW has received: consulting fees from Actelion, Astellas, bioMerieux, Cambimune, Da Volterra, Ferring, MedImmune, Menarini, Merck, Meridian, Pfizer, Qiagen, Sanofi-Pasteur, Seres, Spero, Summit, Synthetic Biologics, and Valneva; lecture fees from Alere, Astellas, Merck, and Pfizer; and grant support from Actelion, Astellas, bioMerieux, Da Volterra, Merck, Motif Biosciences, Nabriva, Paratek, Pfizer, Sanofi-Pasteur, Seres, and Summit. The remaining author declares that the research was conducted in the absence of any commercial or financial relationships that could be construed as a potential conflict of interest. The reviewer SJ declared a past collaboration with one of the authors MW to the handling Editor.
